# A gene expression-based classifier for HER2-low breast cancer

**DOI:** 10.1038/s41598-024-52148-7

**Published:** 2024-02-01

**Authors:** Serena Di Cosimo, Sara Pizzamiglio, Chiara Maura Ciniselli, Valeria Duroni, Vera Cappelletti, Loris De Cecco, Cinzia De Marco, Marco Silvestri, Maria Carmen De Santis, Andrea Vingiani, Biagio Paolini, Rosaria Orlandi, Marilena Valeria Iorio, Giancarlo Pruneri, Paolo Verderio

**Affiliations:** 1 Department of Advanced Diagnostics, Fondazione IRCCS Istituto Nazionale Dei Tumori, Milan, Italy; 2 Bioinformatics and Biostatistics Unit, Department of Epidemiology and Data Science, Fondazione IRCCS Istituto Nazionale Dei Tumori, Milan, Italy; 3 Molecular Mechanisms Unit, Department of Research, Fondazione IRCCS Istituto Nazionale Dei Tumori, Milan, Italy; 4 Radiation Oncology 1, Fondazione IRCCS Istituto Nazionale Dei Tumori, Milan, Italy; 5 Breast Unit, Fondazione IRCCS Istituto Nazionale Dei Tumori, Milan, Italy; 6 Department of Pathology, Fondazione IRCCS Istituto Nazionale Dei Tumori, Milan, Italy; 7 Molecular Targeting Unit, Department of Research, Fondazione IRCCS Istituto Nazionale Dei Tumori, Milan, Italy

**Keywords:** Cancer, Oncology

## Abstract

In clinical trials evaluating antibody-conjugated drugs (ADCs), HER2-low breast cancer is defined through protein immunohistochemistry scoring (IHC) 1+ or 2+ without gene amplification. However, in daily practice, the accuracy of IHC is compromised by inter-observer variability. Herein, we aimed to identify HER2-low breast cancer primary tumors by leveraging gene expression profiling. A discovery approach was applied to gene expression profile of institutional INT1 (n = 125) and INT2 (n = 84) datasets. We identified differentially expressed genes (DEGs) in each specific HER2 IHC category 0, 1+, 2+ and 3+. Principal Component Analysis was used to generate a HER2-low signature whose performance was evaluated in the independent INT3 (n = 95), and in the publicly available TCGA and GSE81538 datasets. The association between the HER2-low signature and HER2 IHC categories was evaluated by Kruskal–Wallis test with post hoc pair-wise comparisons. The HER2-low signature discriminatory capability was assessed by estimating the area under the receiver operating characteristic curve (AUC). Gene Ontology and KEGG analyses were performed to evaluate the HER2-low signature genes functional enrichment. A HER2-low signature was computed based on HER2 IHC category-specific DEGs. The twenty genes included in the signature were significantly enriched with lipid and steroid metabolism pathways, peptidase regulation, and humoral immune response. The HER2-low signature values showed a bell-shaped distribution across IHC categories (low values in 0 and 3+; high values in 1+ and 2+), effectively distinguishing HER2-low from 0 (*p* < 0.001) to 3+ (*p* < 0.001). Notably, the signature values were higher in tumors scored with 1+ as compared to 0. The HER2-low signature association with IHC categories and its bell-shaped distribution was confirmed in the independent INT3, TCGA and GSE81538 datasets. In the combined INT1 and INT3 datasets, the HER2-low signature achieved an AUC value of 0.74 (95% confidence interval, CI 0.67–0.81) in distinguishing HER2-low vs. the other categories, outperforming the individual *ERBB2* mRNA AUC value of 0.52 (95% CI 0.43–0.60). These results represent a proof-of-concept for an observer-independent gene-expression-based classifier of HER2-low status. The herein identified 20-gene signature shows promise in distinguishing between HER2 0 and HER2-low expressing tumors, including those scored as 1+ at IHC, and in developing a selection approach for ADCs candidates.

## Introduction

Assessment of Human Epidermal growth factor Receptor 2 (HER2) status is a standard practice in breast cancer diagnostics^1^. Early clinical studies have indicated that anti-HER2 therapy provides benefit exclusively to patients with breast cancer scoring 3+ at immunohistochemistry (IHC) and/or exhibiting gene amplification at in situ hybridization (ISH)^1–3^. This has resulted in distinguishing treatable HER2-positive tumors from the rest, which are considered negative when IHC scores are 0, 1+, or 2+ without amplification^1^. However, emerging evidence is challenging this binary classification, as antibody–drug conjugates (ADCs)^4^, particularly trastuzumab deruxtecan (T-DXd), showed benefit beyond HER2-positive cases^5^. For instance, in the DESTINY-Breast04 study, T-DXd demonstrated a 50% reduction in the risk of progression, and a 36% reduction in the risk of death in patients with HER2-low (IHC 1+, and 2+ without amplification) metastatic breast cancer (MBC)^6^. This has prompted further investigation in the ongoing clinical trial DESTINY-Breast06^7^, which is evaluating T-DXd in patients with even lower HER2 expression in MBC, specifically IHC 0 or 1+, referred to as ultralow based on the preliminary data of DAISY study showing a 30% clinical response in advanced treatment lines^8^.

While these findings generated enthusiasm for T-DXd as a new effective therapy, concerns arose regarding the reproducibility of HER2-low definition from clinical trials to daily practice^9^. Until now, pathologists have not needed to differentiate between IHC 0, with incomplete and faint staining in ≤ 10% of tumor cells, and IHC 1+, same staining intensity in > 10% of tumor cells. Nor have they been required to distinguish IHC 1+ from 2+ showing weak to moderate staining. However, interpreting IHC results in tumors without over-expression is challenging^10^. A recent study by Fernandez et al.^11^ found the highest rate of discordant cases in IHC between the 0 and 1+ categories, with an agreement reaching 70% in the best-case scenario. This finding was strengthened by the Yale University report, where only 26% of cases showed a 90% agreement in the same categories, and by Schettini et al*.*^12^, who alerted that over half of the IHC discordant cases occurred among HER2-low tumors. Therefore, the routine use of IHC for determining HER2-low status, and assigning new ADC-based therapies raises concerns about analytical validity. Furthermore, it is crucial to investigate the biology of breast cancer falling within the gray zone of IHC+ 1 and 2+ to characterize breast cancer patients who benefit from new therapies.

Gene expression profiling is an undeniable tool for breast cancer characterization and classification^13,14^, and offers advantages over conventional diagnostics including a quantitative output that reduces subjective interpretations allowing a standardized and automated data analysis. Herein, we aimed to analyze breast cancer gene expression profile (GEP) with respect to HER2 IHC categories, and to train a genomic classifier for identifying HER2-low tumors.

## Materials and methods

### Datasets

GEPs from primary tumors of women with newly diagnosed operable breast cancer were retrieved from our institutional internal database (INT). Three distinct datasets with available patient, primary tumor and gene expression information were identified, specifically INT1 (n = 125) and INT2 (n = 84), which served as discovery sets; and INT3 (n = 95), which served as an independent confirmatory set. For each dataset, we accessed patient age, primary tumor size, grade, hormone receptor (HR) status, HER2 according to IHC 0, 1+, 2+, and 3+ categories; and primary tumor GEP at baseline (before any systemic treatment). Detailed information on RNA extraction and quality control has been described in original studies generating gene expression data^15–17^. Briefly, GEP was obtained from RNA extracted from formalin-fixed paraffin-embedded tissue using Affymetrix U133 Plus 2.0 (INT1) and Affymetrix HTA-2.0 platforms (INT2); and from frozen tissue using Illumina Human HT-12_V3.0 platform (INT3). For in silico analysis, we used The Cancer Genome Atlas (TCGA) data from the publicly available TCGA Research Network (http://cancergenome.nih.gov, lastly accessed on September 2022) and the GSE81538^18^ from the NCBI Gene Expression Omnibus (https://www.ncbi.nlm.nih.gov/geo) with RNA sequencing data of 405 breast cancer patients. This study was conducted in accordance with the Declaration of Helsinki. All participants in the original studies had signed an informed consent that allowed the use of their samples for future biomarker research. The original studies were approved by the Institutional Review Board and the Ethics Committee of INT.

### Statistical analysis

Starting from INT1 and INT2 datasets, a filtering procedure was applied to select relevant genes, namely those with a log2 fold change (FC) value greater than 1 in absolute value (|log2(FC)|> 1) by comparing the different IHC categories, specifically 0 versus [vs.] others, 1+ versus others, 2+ versus others, 3+ versus others, and 1+ and 2+ (HER2-low) vs. others. Afterwards, the expression values of the relevant genes were compared across the above categories using the non-parametric Wilcoxon test. Only genes with a statistically significant Bonferroni-adjusted *p* value were considered as significantly differentially expressed. A Venn diagram approach was used to select differentially expressed genes (DEGs) in the specific IHC categories of interest. These category-specific DEGs were then processed by Principal Component Analysis (PCA)^19^ starting from the correlation matrix. The first Principal Component (1st PC) was used to generate a score (HER2-low signature) for each patient by weighting the standardized expression of the IHC category-specific DEGs with the pertinent coefficients. Partial correlations coefficient (r_p_) between the genes of the signature were estimated in order to investigate the correlation between two genes corrected for presence of all the other genes of the signature. The capability of the signature to distinguish HER2-low breast cancer from HER2 0 or 3+ was initially evaluated in the discovery setting, represented by INT1, as INT2 lacked the categories 2+ and 3+, using the non-parametric Kruskal–Wallis (KW) test with post hoc pair-wise comparisons between the IHC category of interest^20^. This evaluation was subsequently extended to the confirmatory INT3, TCGA and GSE81538 datasets. The discriminatory capability of the signature was analysed in terms of area under the receiver operating characteristic (ROC) curve (AUC) with the corresponding 95% confidence interval (CI)^21^. For exploratory purpose, a cut-off value was identified by maximising the Youden index. All statistical analyses were conducted using SAS (version 9.4; SAS Institute, Inc.) and RStudio (version 4.2.1; The R Foundation for Statistical Computing), with a nominal alpha level of 5%.

Functional enrichment of the HER2-low signature for Gene Ontology (GO) biological process terms and KEGG pathways^22^ was performed using the Cluster Profiler Bioconductor package. In particular, only statistically significant enriched pathways (*p* value < 0.05) defined by at least two IHC category-specific DEGs were considered for the analysis. Network representation to evaluate pathways commonalities was performed using Cytoscape 3.8.2 version^23^.

### Ethical approval and consent to participate

This study was conducted in accordance with the Declaration of Helsinki. All participants in the original studies had signed an informed consent that allowed the use of their samples for future biomarker research.

## Results

### Patient characteristics

The overall and dataset-specific characteristics of the 304 study patient population from institutional datasets are reported in Table 1. In INT1, the median age was 49 years (range 26–67 years), and most tumors were between 2 and 5 cm in size (82%), HR-positive (74%), and moderately differentiated, G2 (59%). In INT2, the median age was 53 years (range 26–87 years), all tumors were triple negative, with most being > 5 cm (96%) and undifferentiated, G3 (87%). In INT3, the median age was 60 years (range 35–86 years), tumors ≤ 2 or between 2 and 5 cm had a similar proportion (51% and 44%, respectively), as well as grading, G2 (48%) and G3 (52%), while HR-positive cases were the majority (82%). Figure S1 reports study dataset HER2 IHC categories and hormone receptor status.

**Table 1:** Clinico-pathological characteristics of breast cancer patients in the study datasets.

Clinico-pathological characteristics	Discovery set		Confirmatory set	Total n = 304
	INT1	INT2	INT3	
	n = 125	n = 84	n = 95	
	n (%)	n (%)	n (%)	n (%)
Age (years)				
Median (Range)	49 (26–67)	53 (26–87)	60 (35–86)	52 (26–87)
Missing	–	1	–	1
Tumor size				
≤ 2 cm	16 (13%)	1 (1%)	48 (51%)	65 (21%)
2–5 cm	103 (82%)	1 (1%)	42 (44%)	146 (48%)
> 5 cm	5 (4%)	81 (96%)	4 (4%)	90 (30%)
Missing	1 (1%)	1 (1%)	1 (1%)	3 (1%)
Grade				
II	74 (59%)	11 (13%)	46 (48%)	131 (43%)
III	51 (41%)	73 (87%)	49 (52%)	173 (57%)
Hormone receptors				
Positive	92 (74%)	0 (0%)	78 (82%)	170 (56%)
Negative	30 (24%)	84 (100%)	17 (18%)	131 (43%)
Missing	3 (2%)	–	–	3 (1%)
HER2 IHC				
0	33 (26%)	52 (62%)	25 (26%)	110 (36%)
1+	62 (50%)	32 (38%)	26 (27%)	120 (40%)
2+	12 (10%)	0 (0%)	30 (32%)	42 (14%)
3+	18 (14%)	0 (0%)	14 (15%)	32 (10%)
PAM50 subtype*				
Luminal A	41 (33%)	8 (10%)	48 (51%)	97 (32%)
Luminal B	23 (18%)	10 (12%)	14 (15%)	47 (16%)
HER2-enriched	21 (17%)	4 (5%)	12 (13%)	37 (12%)
Basal-like	19 (15%)	59 (70%)	15 (16%)	93 (31%)
Normal-like	21 (17%)	3 (4%)	0 (0%)	24 (8%)
Undetermined	0 (0%)	0 (0%)	6 (6%)	6 (2%)

*As reported by Prat A, et al. Clin Cancer Res 2014;20:511–521.

Overall, as reported in Table 2, 162 (53%) tumors were HER2-low, of which 119 HR-positive (74%) and 41 HR-negative (25%), and 2 with missing information (1%); 110 were HER2 0 (36%), 31 HR-positive (28%) and 78 HR-negative (71%), and 1 with missing information (1%); 32 were HER2 3+, 20 HR-positive (63.5%), and 12 HR-negative (37.5%). The majority of HER2-low tumors were classified as luminal A (43%) or B (17%).

**Table 2:** Patient characteristics according to HER2 IHC categories.

Patient characteristics	HER2 IHC categories						Total	
	HER2 0		HER2-low		HER2 3+			
	n	%	n	%	n	%	n	%
Tumor size								
≤ 2 cm	13	12	42	26	10	31	65	21
2–5 cm	44	40	83	51	19	59	146	48
> 5 cm	53	48	34	21	3	9	90	30
Missing	0	0	3	2	0	0	3	1
Grade								
II	36	33	86	53	9	28	131	43
III	74	67	76	47	23	72	173	57
Hormone receptors								
Positive	31	28	119	73	20	63	170	56
Negative	78	71	41	25	12	38	131	43
Missing	1	1	2	1	0	0	3	1
PAM50 subtype*								
Luminal A	23	21	69	43	5	16	97	32
Luminal B	18	16	27	17	2	6	47	15
HER2-enriched	4	4	10	6	23	72	37	12
Basal-like	56	51	36	22	1	3	93	31
Normal-like	6	5	18	11	0	0	24	8
Undetermined	3	3	2	1	1	3	6	2
Total	110	100	162	100	32	100	304	100

*As reported by Prat A, et al. Clin Cancer Res 2014;20:511–521.

The overall distribution of PAM50 subtypes according to HER2 IHC categories and HR status is reported in Table S1.

### HER2 IHC category-specific genes and HER2-low signature

**Figure 1:**
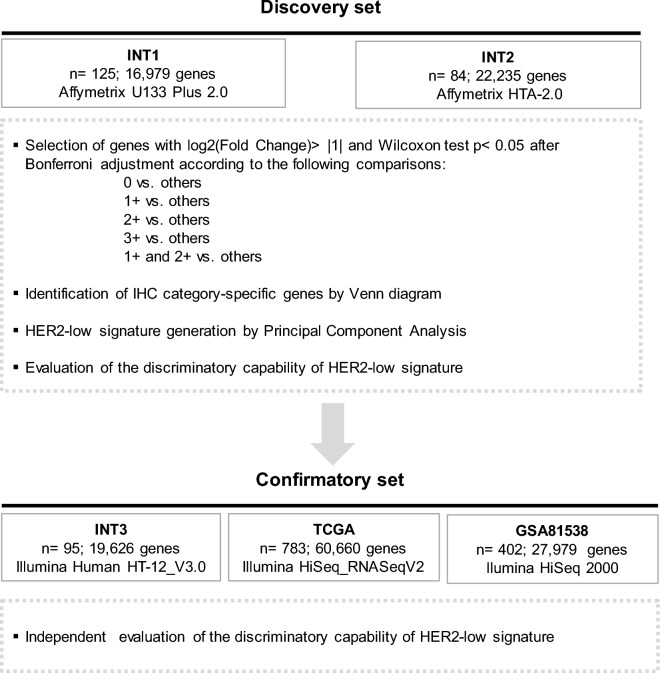
Study flowchart for HER2-low signature development and testing.

**Figure 2:**
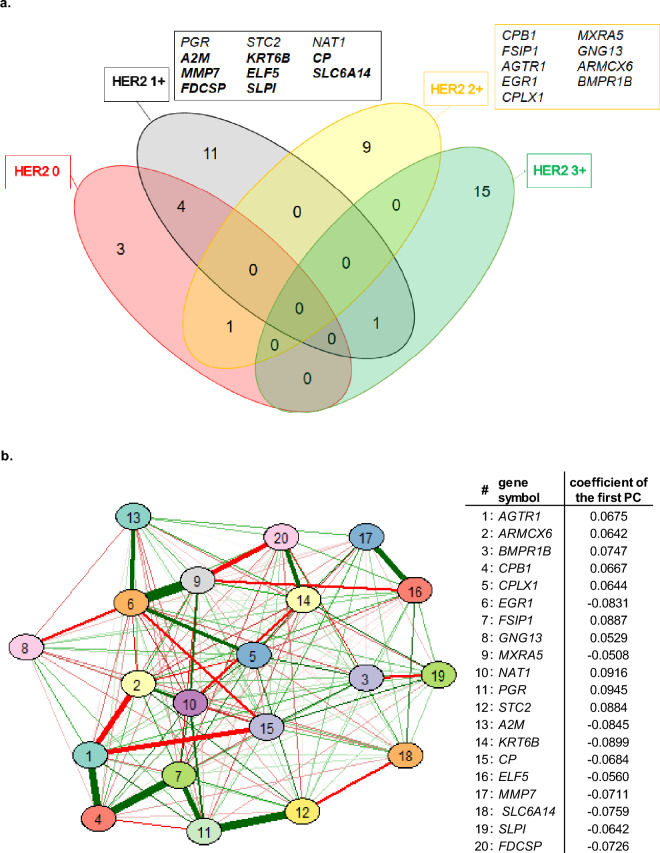
Identification of the 20 IHC category-specific genes and development of the HER2-low signature. a. The Venn diagram represents the number of specific and common genes for each HER2 IHC category, following their selection by the fold change and Wilcoxon test. The 12 genes identified in the INT1 dataset are in thin (3 genes in 1+, 8 genes in 2+ and one gene (*CPLX1*) in HER2-low); the 8 genes identified in the INT2 dataset are in bold; b. Partial correlation diagram and coefficient of the first principal component (PC) for the 20 IHC category-specific genes. The full order partial correlations between the 20 genes are visualized as a network, in which nodes represent genes and edges the dependencies between them. Thicker lines indicate higher correlation, with green lines representing positive correlations between genes and red lines indicating negative correlations. The HER2-low signature value was computed by combining the standardized expression of each gene with the pertinent first PC coefficient reported in the right part of the panel.

HER2 DEGs according to the IHC category were identified following the study workflow shown in Fig. 1. A set of 20 IHC category-specific DEGs was selected, 11 genes in 1+, 8 genes in 2+ and 1 gene (*CPLX1*) in the HER2-low category (Fig. 2, panel a). The 1st PC was used to define the HER2-low signature values by combining the standardized expression of each IHC category-specific DEGs with the pertinent coefficient (Fig. 2, panel b). Among the HER2-low signature genes, the highest correlation (partial correlation coefficient, r_p_ = 0.41) was observed between *EGR1* and *MXRA5*, followed by *AGTR1* and *CPB1* (r_p_ = 0.36), *PGR* and *STC2* (r_p_ = 0.35) and *CPB1* and *FSIP1* (r_p_ = 0.34) (Fig. 2, panel b). No other correlations with an absolute value of r_p_ greater than 0.30 were found suggesting that each gene within the HER2-low signature carries its own information.

### HER2-low gene signature discriminatory capability

**Figure 3:**
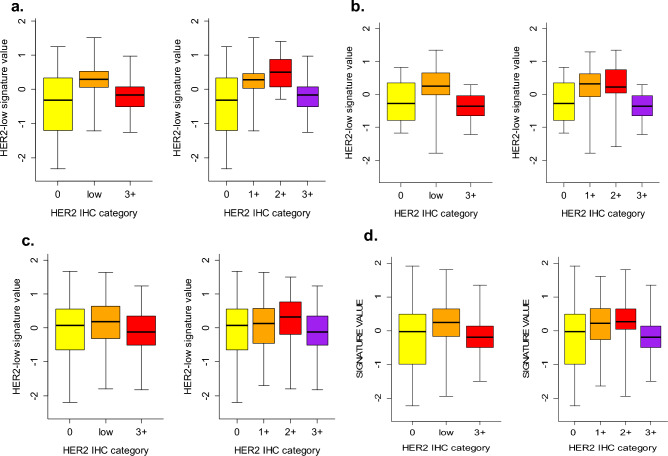
Distribution of HER2-low signature values by HER2 IHC categories. a. Discovery; b. Confirmatory; and publicly available datasets, c.TCGA and d. GSE81538. Graphics report the distribution of the HER2-low signature values according to HER2 IHC categories: 0, HER2-low, and 3+ (left side); and 0, 1+, 2+, 3+ (right side). Each box indicates the 25th and 75th percentile. The horizontal lines inside the box indicate the median, whiskers indicate the extreme values. Discovery data set, excluding INT2 due to the absence of all IHC categories: HER2-low versus 0 (*p* = 0.0002) and versus 3+ (*p* = 0.0009); Confirmatory data set: HER2-low versus 0 (*p* = 0.0262) and versus 3+ (*p* = 0.0018); TCGA data set: HER2-low versus 0 (*p* value 0.049) and versus 3+ (*p* value 0.003) and GSE81538 data set: HER2-low versus 0 (*p* value 0.0005) and verus 3+ (*p* value < 0.0001).

In the discovery set, the HER2-low signature values were significantly associated with HER2 IHC categories (KW *p* < 0.0001) and exhibited a bell-shaped distribution across IHC categories, distinguishing HER2-low from both 0 (*p* = 0.0002) and 3+ (*p* = 0.0009) (Fig. 3, panel a). These findings were confirmed in the independent INT3 dataset, where a statistically significant association was found between the HER2-low signature and the IHC categories (KW *p* = 0.0008). Specifically, HER2-low signature values were higher in HER2-low compared to 0 (*p* = 0.0262) and 3+ (*p* = 0.0018) (Fig. 3, panel b). To further confirm these findings, we analyzed two publicly available datasets, TCGA and GSE81538 which included 783 and 402 breast cancer cases (Table S2). Once again, there was a significant association between the HER2-low signature and IHC categories (TGCA KW *p* = 0.0017 and GSE81538 KW *p* < 0.0001), with higher values in HER2-low compared to 0 (TCGA *p* = 0.049 and GSE81538 *p* < 0.0001) and 3+ (TCGA *p* = 0.003 and GSE81538 *p* = 0.0005) (Fig. 3, panel c and d).

Notably, across all the explored datasets the HER2-low signature consistently displayed its highest values within the 1+ and 2+ categories. While the HER2-low signature values exhibited a distribution pattern in HR-positive cases similar to that of the overall population in each dataset, the limited number of HR-negative cases precluded any conclusive analysis (Fig. S2). However, in the largest TCGA dataset, with 69 out of 320 (18%) cases classified as HR-negative/HER2-low, the signature values showed higher levels in HER2-low compared to the 0 category (*p* = 0.02) (Fig. S2, panel c).

### HER2-low signature, ERBB2 mRNA levels and PAM50 subtypes

In the combined discovery and confirmatory dataset, the HER2-low signature achieved an AUC value of 0.74 (95% CI 0.67–0.81) in distinguishing HER2-low vs. the other categories, outperforming the individual *ERBB2* mRNA AUC value of 0.52 (95% CI 0.43–0.60).

HER2-low signature values were higher in luminal A and B compared to other subtypes (*p* < 0.0001) (Fig. S3), consistent with 71% of HER2-low cases being classified as luminal A or B (Table S3). It is worth noting that high signature levels, i.e. above the Youden cut-off, effectively captured 45% of HER2-low cases with non-luminal-like features (Fig. S3, panel b).

### HER2-low signature gene enrichment analysis

Gene Ontology and KEGG pathway analyses were finally conducted showing significant alterations in pathways related to Gene Ontology, particularly in the biological process category. HER2-low signature genes showed common enrichment in lipid and steroid metabolic processes, regulation of peptidase, and humoral immune response. The network analysis revealed distinct interconnected patterns among the genes, with the most significant functional modules involving *EGR1* and *STC2* and associated with oxygen regulation, metabolic processes, serine/threonine kinase signaling, and immune response (Fig. S4).

## Discussion

This study represents the first report leveraging a gene expression-based method for HER2-low assessment. By analyzing mRNA profiles from 304 breast cancer patients, a set of genes specifically associated with IHC categories was integrated into a signature capable of distinguishing HER2-low from both HER2 0 and 3+ tumors. The findings were validated through in silico analysis. Additionally, the study provides insights into the clinico-pathological features and distribution of intrinsic subtypes of HER2-low breast cancer using publicly available datasets as references, while also exploring their underlying biology.

HER2 assessment is of growing importance in breast cancer management, as clinical trials of HER2-targeted ADCs have demonstrated promising outcomes in patients with HER2-low breast cancer^4–8^. However, the identification of these tumors in routine clinical practice remains challenging as the IHC-based evaluation lacks optimal inter-observer concordance, especially in the 0 and 1+ categories^11,12^. Building upon the hypothesis that mRNA expression has a wide dynamic range, recent studies have explored quantitative *ERBB2* mRNA levels as a means to develop a new assay for HER2-low breast cancer. However, Xu et al.^24^ reported low concordance between IHC and quantitative RT-PCR methods for evaluating HER2 status, and Shu et al. confirmed these findings, especially in HER2 0 and 1+ cases^25^. Both studies concluded that single gene expression is inconclusive and unable to distinguish between HER2-low and HER2 0 categories. Another attempt to apply gene expression analysis was based on the hypothesis that HER2-low tumors could be HER2-enriched^26^. However, only a small fraction of these tumors fits this criteria; in our study, 7% of HER2-low/HR-positive and 5% of HER2-low/HR-negative cases, which is in line with the literature^12,26^. At variance with previous studies based on evaluating single *ERBB2* mRNA and/or its targeted genes, we herein employed a distinct discovery-based approach to identify the most informative transcriptomic features for each HER2 IHC category by maximizing their differences. These features were then integrated into a scoring system. Across all the explored datasets, the HER2-low signature consistently displayed its lowest values within the 0 category. The findings highlight the significance of combining multiple transcriptomic features to improve their efficacy and address limitations associated with both existing genomic classifiers and single gene expression assessment methods. Moreover, a gene expression-based HER2 classifier offers advantages as an additional test in challenging cases, complementing existing IHC evaluation, and provides observer independence and cost-effectiveness, particularly in resource-limited situations.

The need to accurately identify HER2-low, particularly HER2 1+, arises with the advent of antibody–drug conjugates in clinical practice. A tool immune to IHC variability is crucial to ensure effective treatment and avoid unnecessary toxic and expensive interventions. The cost-effectiveness of current genomic classifiers, such as RT-PCR and open array techniques, further supports their suitability for this purpose.

At the time the present study was conducted, ESMO expert consensus statements advised considering HER2-low breast cancer as a heterogeneous disease primarily influenced by hormone receptor expression rather than a distinct molecular entity^27^. This advice was based on previous studies that failed to identify consistent and specific differences in mutational profiles between HER2-low and HER2 0 tumors^28^. Similarly, minimal differences in intrinsic subtype classification have been observed once hormone receptors were taken into account^12,26^, as reported in our study as well.

In this context, our findings offer some insights into HER2-low breast cancer biology. Firstly, we observed higher HER2-low signature values in HR-positive breast cancer. While transcript levels may not directly reflect protein expression or activity, it is noteworthy that among the HER2-low signature genes, *EGR1*and *STC2*, which are known estrogen receptor targets, have emerged as key players with significant functional roles. Prior breast cancer studies reported down-regulation of *EGR1* is linked to unfavorable prognosis^29^, while up-regulation of *STC2* reduces cell proliferation, inhibits epithelial-mesenchymal transition, and is associated with late rather than early recurrence^30^.

Secondly, the HER2-low signature includes genes that are outside the ER transcriptional network, previously described in breast cancer or other malignancies, and with a potential as molecular targets. Among these, *SLPI* (Secretory Leukocyte Protease Inhibitor) is involved in inflammation inhibition, immune response modulation, and cell proliferation promotion^31^, and is found to be elevated in triple-negative breast cancer patients with poor prognosis^32^. *A2M* (Alpha-2-Macroglobulin) has been shown to affect adhesion, migration, and growth by inhibiting signaling pathways such as PI3K/AKT and SMAD, while also increasing PTEN levels by down-regulating miR-21^33^. *AGTR1* is a poorly described gene in breast cancer that has recently gained attention as a potential target for drug repurposing^34^. Lastly, the HER2-low signature appears to perform equally well in identifying HER2-low cases in both HR-positive and HR-negative tumors, although the low number of HR-negative cases prevented conclusive analyses. Since HER2-low cases with HR-negative status are uncommon^12,26^, further confirmatory analysis using a larger dataset is required.

The strengths of the present study lie in the sufficiently large patient cohorts from a single center. All patients participated in a clinical trial, enhancing the reliability of our findings. Patient primary tumor tissues underwent gene expression profiling using microarray technology. Importantly, the HER2-low signature performance was consistent across various microarray platforms and in silico analysis, indicating its independence from technical aspects and probe quality. However, there are certain limitations to consider. This study was conducted on samples that may not be representative of the overall breast cancer population, therefore the signature should be validated in a prospective setting. The study is based on available retrospective data and further research is needed to determine the optimal cut-off for clinical application. Additionally, the verification of the signature relied on publicly available databases, and results are to be confirmed in additional external datasets.

## Conclusion

This study represents a promising proof of concept for the utilization of a gene expression-based classifier in HER2-low breast cancer. The 20-gene HER2-low signature identified here, holds potential especially in distinguishing between HER2 0 and HER2-low expressing tumors, even those scored as 1+ at IHC, and in developing a selection approach for ADC candidates. Additionally, we have identified specific HER2-low genes, particularly those impinging on metabolism and immune response processes, that warrant further investigation and may contribute to defining HER2-lowness in the next future.

### Supplementary Information

Supplementary Information.

## Data Availability

The datasets used and/or analysed during the current study are available from the corresponding author on reasonable request.

## Abbreviations

A2M: Alpha-2-macroglobulin
ACDs: Antibody-conjugated drugs
AUC: Area under the receiver operating characteristic curve
CI: Confidence interval
DEGs: Differentially expressed genes
FC: Fold change
GEP: Gene expression profile
GO: Gene ontology
HER2: Human epidermal growth factor receptor 2
HR: Hormone receptor
IHC: Immunohistochemistry
INT: Institutional internal database
ISH: In situ Hybridization
KW: Kruskal-Wallis
MBC: Metastatic breast cancer
*p*: *p* value
PCA: Principal component analysis
ROC: Receiver operating characteristic
r_p_: Partial correlations coefficient
SLPI: Secretory leukocyte protease inhibitor
TCGA: The cancer genome atlas
T-DXd: Trastuzumab deruxtecan
vs.: Versus

## References

[1] Wolff AC, Hammond MEH, Allison KH, Harvey BE, Mangu PB, Bartlett JMS (2018). Human epidermal growth factor receptor 2 testing in breast cancer: American society of clinical oncology/college of American pathologists clinical practice guideline focused update. J. Clin. Oncol..

[2] Loibl S, Gianni L (2017). HER2-positive breast cancer. Lancet.

[3] Slamon DJ, Clark GM, Wong SG, Holt JA, Wong SG, Keith DE (1989). Studies of the HER-2/neu proto-oncogene in human breast and ovarian cancer. Science.

[4] Rassy E, Rached L, Pistilli B (2022). Antibody drug conjugates targeting HER2: Clinical development in metastatic breast cancer. Breast.

[5] Modi S, Park H, Murthy RK, Iwata H, Tamura K, Tsurutani J (2020). Antitumor activity and safety of trastuzumab deruxtecan in patients with HER2-low-expressing advanced breast cancer: Results from a phase Ib study. J. Clin. Oncol..

[6] Modi S, Jacot W, Yamashita T, Sohn J, Vidal M, Tokunaga E (2022). Trastuzumab deruxtecan in previously treated HER2-low advanced breast cancer. N. Engl. J. Med..

[7] Bardia A, Barrios C, Dent R, Hu X, O’Shaughnessy J, Yonemori K (2021). Trastuzumab deruxtecan (T-DXd; DS-8201) vs investigator’s choice of chemotherapy in patients with hormone receptor-positive (HR+), HER2 low metastatic breast cancer whose disease has progressed on endocrine therapy in the metastatic setting: A randomized, global phase 3 trial (DESTINY-Breast06). Cancer Res..

[8] Mosele F, Deluche E, Lusque A, Le Bescond L, Filleron T, Pradat Y (2023). Trastuzumab deruxtecan in metastatic breast cancer with variable HER2 expression: The phase 2 DAISY trial. Nat. Med..

[9] Rimm, D. HER2 low: A pathologist’s perspective, in *2022 San Antonio Breast Cancer Symposium. Special Session. Presented December 7* (2022).

[10] Sajjadi E, Venetis K, Ivanova M, Fusco N (2022). Improving HER2 testing reproducibility in HER2-low breast cancer. Cancer Drug Resist..

[11] Fernandez AI, Liu M, Bellizzi A, Brock J, Fadare O, Hanley K (2022). Examination of low ERBB2 protein expression in breast cancer tissue. JAMA Oncol..

[12] Schettini F, Chic N, Brasó-Maristany F, Paré L, Pascual T, Conte B (2021). Clinical, pathological, and PAM50 gene expression features of HER2-low breast cancer. NPJ Breast Cancer.

[13] Golub TR, Slonim DK, Tamayo P, Huard C, Gaasenbeek M, Mesirov JP (1999). Molecular classification of cancer: Class discovery and class prediction by gene expression monitoring. Science.

[14] Perou CM, Sorlie T, Eisen MB, van de Rijn M, Jeffrey SS, Rees CA (2000). Molecular portraits of human breast tumours. Nature.

[15] Lecchi M, Verderio P, Cappelletti V, De Santis F, Paolini B, Monica M (2021). A combination of extracellular matrix- and interferon-associated signatures identifies high-grade breast cancers with poor prognosis. Mol. Oncol..

[16] Romero-Cordoba SL, Rodriguez-Cuevas S, Bautista-Pina V, Maffuz-Aziz A, D'Ippolito E, Cosentino G (2018). Loss of function of miR-342-3p results in MCT1 over-expression and contributes to oncogenic metabolic reprogramming in triple negative breast cancer. Sci. Rep..

[17] Huang X, Dugo M, Callari M, Sandri M, De Cecco L, Valeri B (2015). Molecular portrait of breast cancer in China reveals comprehensive transcriptomic likeness to Caucasian breast cancer and low prevalence of luminal A subtype. Cancer Med..

[18] Brueffer C, Vallon-Christersson J, Grabau D, Ehinger A (2018). Clinical value of RNA sequencing-based classifiers for prediction of the five conventional breast cancer biomarkers: A report from the population-based multicenter sweden cancerome analysis network-breast initiative. JCO Precis. Oncol..

[19] Joliffe T (1986). Principal Component Analysis.

[20] Hollander M, Wolfe DA, Chicken E (1999). Non Parametric Statistical Methods.

[21] Hanley JA, McNeil BJ (1982). The meaning and use of the area under a receiver operating characteristics (ROC) curve. Radiology.

[22] Kanehisa M, Furumichi M, Sato Y, Kawashima M, Ishiguro-Watanabe M (2023). KEGG for taxonomy-based analysis of pathways and genomes. Nucl. Acids Res..

[23] Shannon P, Markiel A, Ozier O, Baliga NS, Wang JT, Ramage D (2003). Cytoscape: A software environment for integrated models of biomolecular interaction networks. Genome Res..

[24] Xu K, Bayani J, Mallon E, Pond GR, Piper T (2022). Discordance between immunohistochemistry and Erb-B2 receptor tyrosine kinase 2 mRNA to determine human epidermal growth factor receptor 2 low status for breast cancer. J. Mol. Diagn..

[25] Shu L, Tong Y, Li Z, Chen X, Shen K (2022). Can HER2 1+ breast cancer be considered as HER2-low tumor? A comparison of clinicopathological features, quantitative HER2 mRNA levels, and prognosis among HER2-negative breast cancer. Cancers (Basel).

[26] Agostinetto E, Rediti M, Fimereli D, Debien V, Piccart M, Aftimos P (2021). HER2-low breast cancer: Molecular characteristics and prognosis. Cancers (Basel).

[27] Tarantino P, Viale G, Press MF, Hu X, Penault-Llorca F, Bardia A (2023). ESMO expert consensus statements (ECS) on the definition, diagnosis, and management of HER2-low breast cancer. Ann Oncol.

[28] Marra A, Safonov A, Drago J, Ferraro E, Selenica P, Gazzo A (2023). Genomic characterization of primary and metastatic HER2-low breast cancers. Cancer Res..

[29] Wang B, Guo H, Yu H, Chen Y, Xu H, Zhao G (2021). The role of the transcription factor EGR1 in cancer. Front. Oncol..

[30] Qie S, Sang N (2022). Stanniocalcin 2 (STC2): A universal tumour biomarker and a potential therapeutical target. J. Exp. Clin. Cancer Res..

[31] Zhang X, Liu SS, Ma J, Qu W (2023). Secretory leukocyte protease inhibitor (SLPI) in cancer pathophysiology: Mechanisms of action and clinical implications. Pathol. Res. Pract..

[32] Xie W, Zhang H, Qin S, Zhang J, Fan X, Yin Y (2019). The expression and clinical significance of secretory leukocyte proteinase inhibitor (SLPI) in mammary carcinoma using bioinformatics analysis. Gene.

[33] Kurz S, Thieme R, Amberg R, Groth M, Jahnke HG, Pieroh P (2017). The anti-tumorigenic activity of A2M-A lesson from the naked mole-rat. PLoS One.

[34] Kumar U, Aich J, Devarajan S (2023). Exploring the repurposing potential of telmisartan drug in breast cancer: an in-silico and in-vitro approach. Anti-cancer Drugs.

