# Supplementary material for: A gene expression-based classifier for HER2-low breast cancer
Source: Sci Rep. 2024 Feb 1;14:2628. doi: 10.1038/s41598-024-52148-7 (PMC10830477; doi:10.1038/s41598-024-52148-7)
Supplement: Supplementary file 1 — Supplementary Information. [file 41598_2024_52148_MOESM1_ESM.pdf]

Table S1 Distribution of PAM50 subtypes according to HER2 IHC categories and hormone receptor status

	Study patient population (n= 304**)													
**PAM50 subtypes***	**HER2 IHC category and HR status**													
	**HER2 0/HR+**		**HER2-low/HR+**		**HER2 3+/HR+**		**HER2 0/HR-**		**HER2-low/HR-**		**HER2 3+/HR-**		**Total**	
	**n**	***(%)***	**n**	***(%)***	**n**	***(%)***	**n**	***(%)***	**n**	***(%)***	**n**	***(%)***	**n**	***(%)***
Luminal A	17	*55*	65	*55*	5	*25*	5	*6*	4	*10*	0	*0*	96	*32*
Luminal B	8	*26*	27	*23*	2	*10*	10	*13*	0	*0*	0	*0*	47	*16*
HER2-enriched	1	*3*	8	*7*	12	*60*	3	*4*	2	*5*	11	*92*	37	*12*
Basal-like	1	*3*	3	*3*	0	*0*	55	*71*	33	*80*	1	*8*	93	*31*
Normal-like	2	*6*	14	*12*	0	*0*	4	*5*	2	*5*	0	*0*	22	*7*
undeterminated	2	*6*	2	*2*	1	*5*	1	*1*	0	*0*	0	*0*	6	*2*
**Total**	31	*100*	119	*100*	20	*100*	78	*100*	41	*100*	12	*100*	301	*100*

HR+, hormone receptor positive; HR-, hormone receptor negative;
* as reported by Prat A, et al. Clin Cancer Res 2014;20:511-521; ** HR status missing in 2 cases classified as HER2-low/normal-like, and 1 case classified as HER2 0/luminal A

Table S2 Clinico-pathological characteristics of breast cancer patients in the TCGA and GSE81538 datasets

	**TCGA n=783**	**GSE81538 n=402**
	**n (%)**	**n (%)**
**Hormone receptors**		
Positive	617 (78.80%)	80 (19.90%)
Negative	166 (21.20%)	322 (80.10%)
**HER2 IHC**		
0	260 (33.2%)	129 (32.09%)
1+	253 (32.3%)	115 (28.61%)
2+	136 (17.4%)	74 (18.41%)
3+	134 (17.1%)	84 (20.90%)
**PAM50 subtype***		
Luminal A	391 (50%)	156 (38.81%)
Luminal B	156 (20%)	103 (25.62%)
HER2-enriched	64 (8.2%)	64 (15.92%)
Basal-like	133 (17.0%)	57 (14.18%)
Normal-like	29 (3.7%)	22 (5.47%)
undetermined	10 (1.3%)	0 (0%)

*as reported by Prat A, *et al*. Clin Cancer Res 2014;20:511-521

Table S3 Distribution of PAM50 subtypes according to HER2 IHC categories in the discovery and confirmatory set

	**HER2 IHC category**									
**PAM50 subtype****	**0**		**1+**		**2+**		**3+**		**total**	
	**n**	***%***	**n**	***%***	**n**	***%***	**n**	***%***	**n**	***%***
Luminal A	19	*33*	45	*51*	20	*48*	5	*16*	89	*40*
Luminal B	8	*14*	17	*19*	10	*24*	2	*6*	37	*17*
HER2-enriched	1	*2*	5	*6*	4	*10*	23	*72*	33	*15*
Basal-like	23	*40*	7	*8*	3	*7*	1	*3*	34	*15*
Normal-like	4	*7*	14	*16*	3	*7*	0	*0*	21	*10*
undetermined	3	*5*	0	*0*	2	*5*	1	*3*	6	*3*
**Total**	58	*100*	88	*100*	42	*100*	32	*100*	220	*100*

Data refer to combined INT 1 and INT 3 (n= 220), which are the datasets containing all the HER2-immunohistochemical categories.

** as reported by Prat A, *et al*. Clin Cancer Res 2014;20:511-521

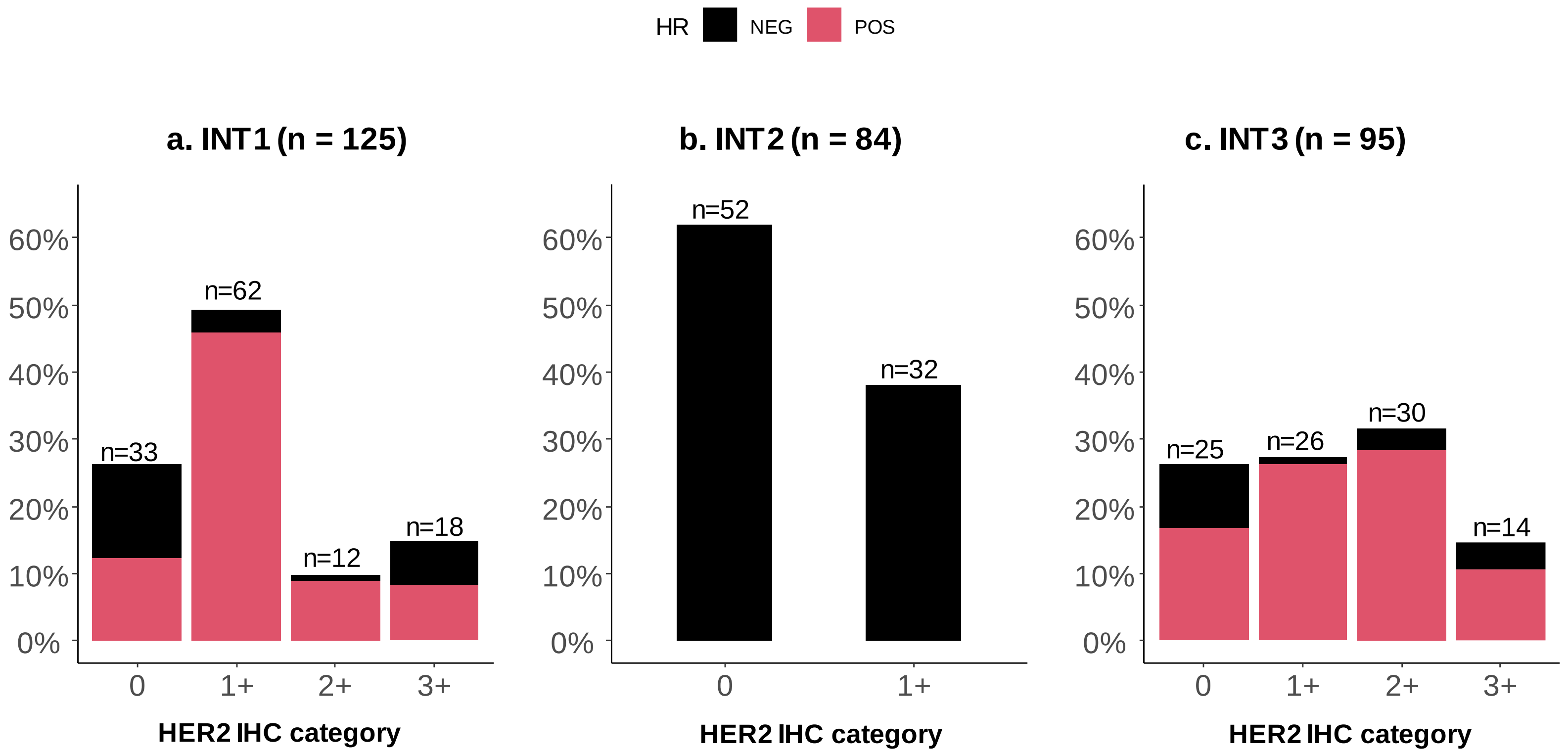

Fig S1 Study dataset HER2 IHC categories and hormone receptor status

Distribution of HR-positive and HR-negative cases in each HER2 IHC category: a. INT1, b.INT2 and c. INT3. HR-positive and HR-negative cases are represented in red and black, respectively. The number of cases for each HER2 IHC category in the study datasets are also reported. HR, hormone receptors

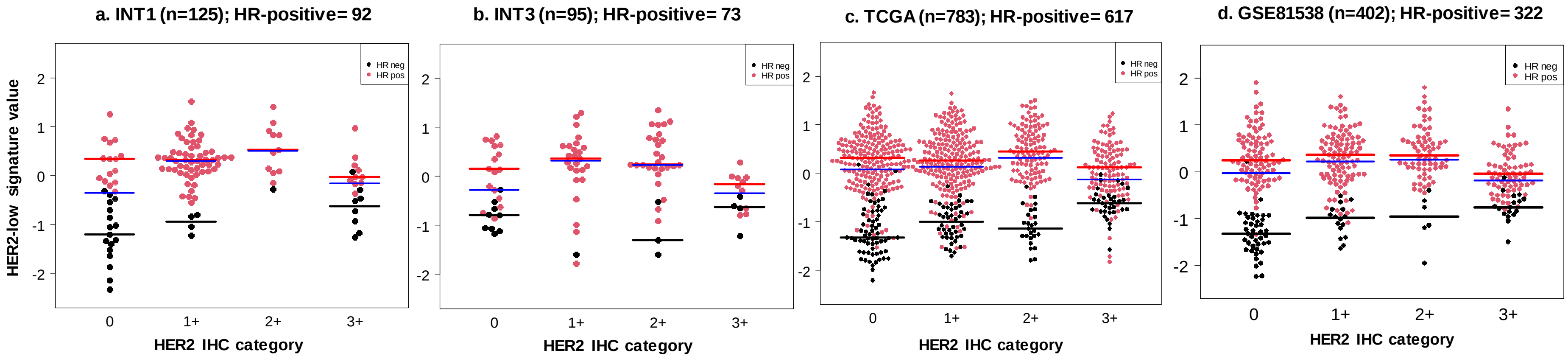

HER2 IHC category	Hormone receptors	
	Positive n	Negative n
0	15	17
1+	56	4
2+	11	1
3+	10	8

HER2 IHC category	Hormone receptors	
	Positive n	Negative n
0	16	9
1+	25	1
2+	27	3
3+	10	4

HER2 IHC categoryv	Hormone receptors	
	Positive n	Negative n
0	196	64
1+	209	44
2+	111	25
3+	101	33

HER2 IHC categoryv	Hormone receptors	
	Positive n	Negative n
0	88	41
1+	99	16
2+	68	6
3+	67	17

Fig S2 Distribution of the HER2-low signature by hormone receptors status

Scatter plots show the distribution of the HER2-low signature values according to IHC categories in HR-positive and HR-negative cases in the study datasets. a. INT1, b.INT2, c. TCGA datasets and d. GSE81538. For each plot, red and black spots represent HR-positive and HR-negative cases, respectively; the horizontal lines represent the median value of the HER2-low signature; specifically, in HR-positive (red line), in HR-negative (black line), and in the entire dataset (blue line). Tables report the number of cases analyzed for each HER2 IHC category according to HR-status. HR, hormone receptors

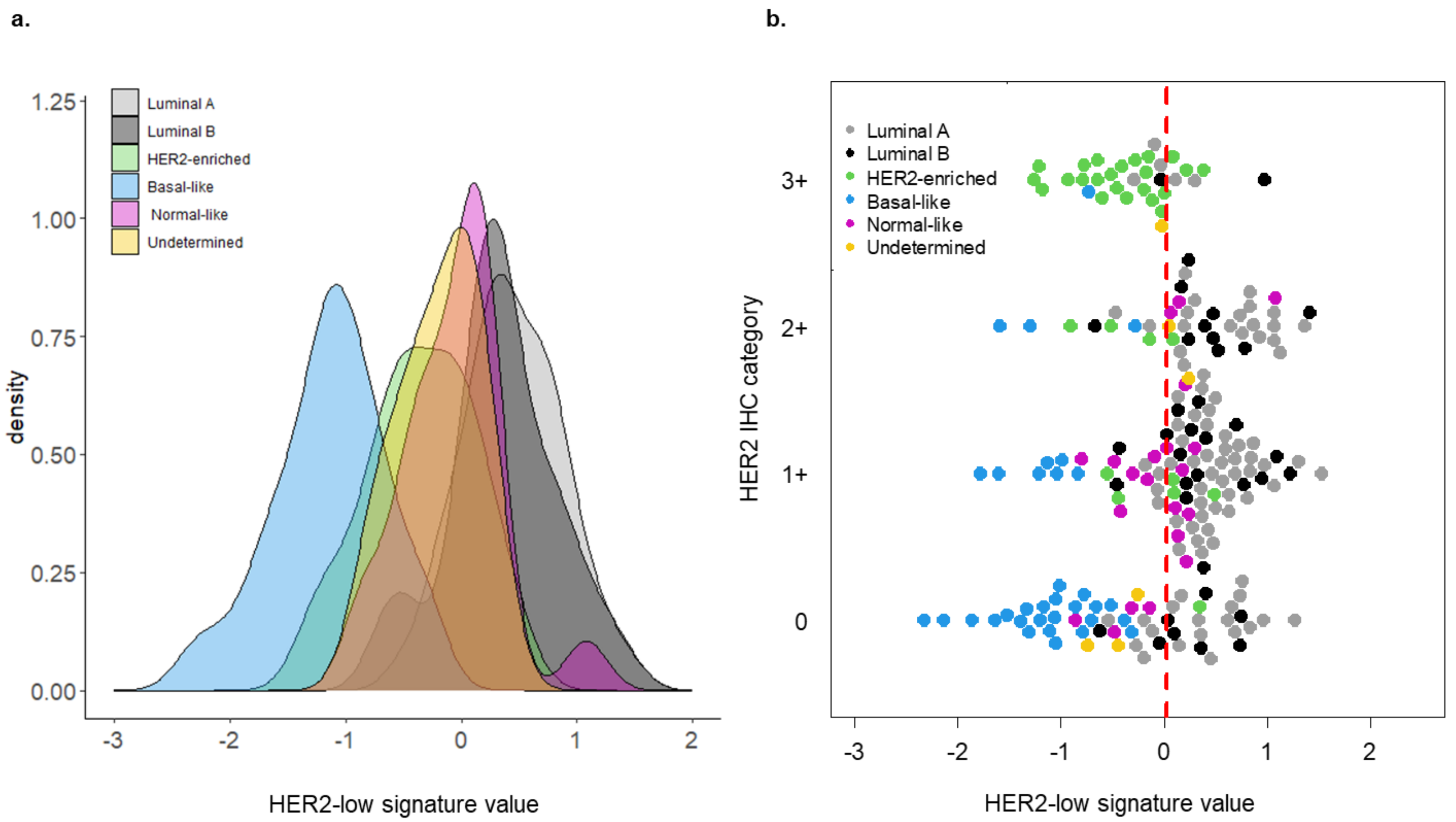

Fig S3 Distribution of the HER2-low signature by breast cancer intrinsic subtypes

a. Density distribution of HER2-low signature by breast cancer intrinsic subtypes. b. Distribution of HER2-low signature by breast cancer intrinsic subtypes and HER2 IHC category. The vertical red dashed line represents the value of the Youden cut-off. Data refer to a combined discovery and institutional confirmatory dataset (n=220), excluding INT2 due to the absence of HR-positive cases and all IHC categories.

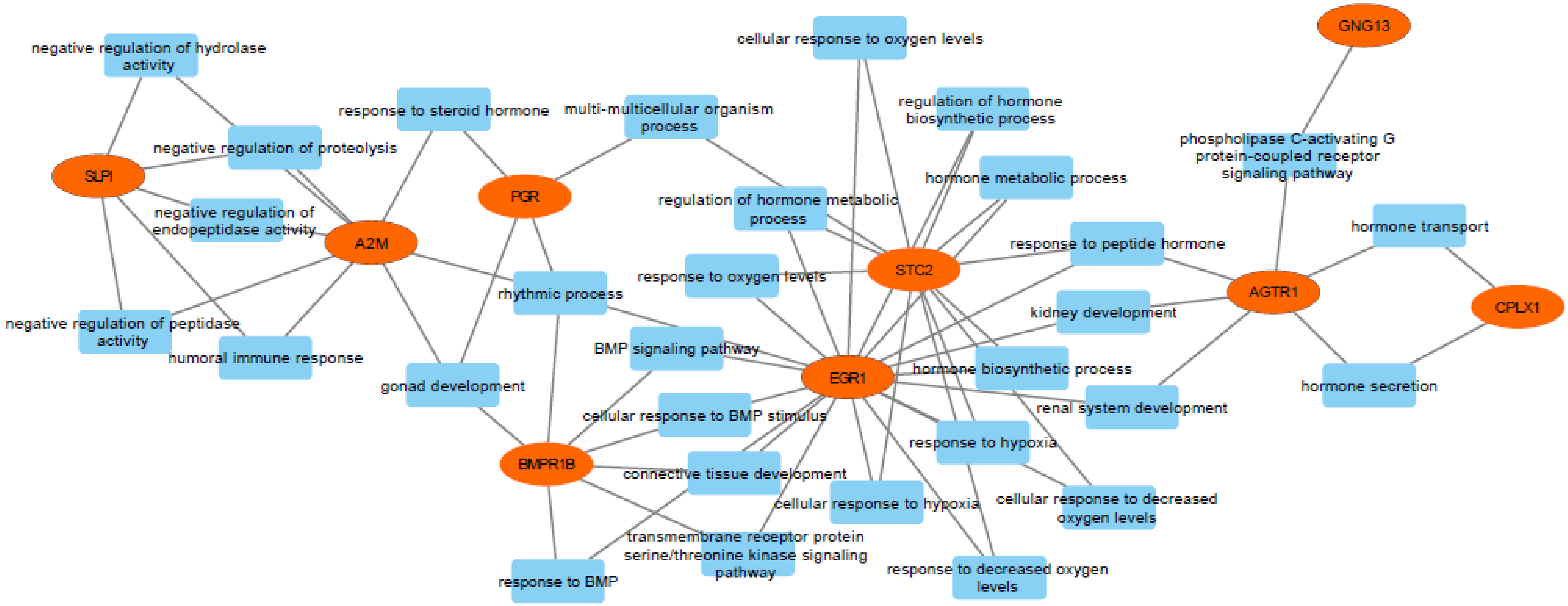

Fig S4 Functional interaction network analysis of HER2-low genes

The graph shows statistically significant pathways sustained by a minimum of two of the twenty gene composing the HER2-low signature.
